# Pre-gelled Electrode Grid for Self-Applied EEG Sleep Monitoring at Home

**DOI:** 10.3389/fnins.2022.883966

**Published:** 2022-06-24

**Authors:** Carlos F. da Silva Souto, Wiebke Pätzold, Marina Paul, Stefan Debener, Karen Insa Wolf

**Affiliations:** ^1^Fraunhofer Institute for Digital Media Technology IDMT, Branch Hearing, Speech and Audio Technology HSA, Oldenburg, Germany; ^2^PSG-Auswertungs-Service, Stadtlohn, Germany; ^3^Neuropsychology Laboratory, Department of Psychology, University of Oldenburg, Oldenburg, Germany; ^4^Cluster of Excellence Hearing4All, University of Oldenburg, Oldenburg, Germany

**Keywords:** EEG, sleep staging, grapho-elements, flexible PCB electrodes, self-application

## Abstract

The need for diagnostic capabilities for sleep disorders such as sleep apnea and insomnia far exceeds the capacity of inpatient sleep laboratories. Some home monitoring systems omit electroencephalography (EEG) because trained personnel may be needed to apply EEG sensors. Since EEG is essential for the detailed evaluation of sleep, better systems supporting the convenient and robust recording of sleep EEG at home are desirable. Recent advances in EEG acquisition with flex-printed sensors promise easier application of EEG sensor arrays for chronic recordings, yet these sensor arrays were not designed for sleep EEG. Here we explored the self-applicability of a new sleep EEG sensor array (trEEGrid) without prior training. We developed a prototype with pre-gelled neonatal ECG electrodes placed on a self-adhesive grid shape that guided the fast and correct positioning of a total of nine electrodes on the face and around the ear. Positioning of the sensors was based on the results of a previous ear-EEG sleep study (da Silva Souto et al., 2021), and included electrodes around the ear, one eye, and the chin. For comparison, EEG and electrooculogram channels placed according to the American Academy of Sleep Medicine criteria, as well as respiratory inductance plethysmography on thorax and abdomen, oxygen saturation, pulse and body position were included with a mobile polysomnography (PSG) system. Two studies with 32 individuals were conducted to compare the signal quality of the proposed flex-printed grid with PSG signals and to explore self-application of the new grid at home. Results indicate that the new array is self-applicable by healthy participants without on-site hands-on support. A comparison of the hypnogram annotations obtained from the data of both systems revealed an overall substantial agreement on a group level (Cohen’s κ = 0.70 ± 0.01). These results suggest that flex-printed pre-gelled sensor arrays designed for sleep EEG acquisition can facilitate self-recording at home.

## Introduction

Sleep disorders are on the rise (Jahrami et al., 2021). Traditionally, a sleep laboratory has been the best diagnostic tool to identify symptoms concerning sleep. However, many sleep disorders are evasive to one night of disrupted sleep in a laboratory setting, which may lead to over- or underdiagnosing of symptoms. Typical sleep disorders like insomnia or sleep bruxism may not be correctly identified in a single night of sleep, but only over time (Miettinen et al., 2017). Similarly, gradual changes in sleep architecture associated with neurological diseases like Parkinson’s disease (Figorilli et al., 2020) or Alzheimer’s disease (Mander, 2020), or mental disorders like depression (Paunio et al., 2015; Goldstone et al., 2020) cannot be detected easily in an environment that is set up for a finite diagnosis after one night. Reliable diagnostic tools are needed that can be used long-term at home with minimal invasion to a patient’s comfort and rest.

If the sleep laboratory is to be considered the highest level of sleep monitoring (type I sleep measurement, typification based on Miettinen et al., 2017), the next level is the preparation of sensors at a sleep center, after which patients are sent home to sleep (type II sleep measurement). While this second level may give professionals more control over data quality, having participants or patients come into a center to prepare for unattended at-home measurements has shown failure rates that are comparable to self-applied unattended at-home measurements (type III sleep measurement). Reliable type III sleep measurements are desirable, however. In the light of the ongoing Covid-19 pandemic, the focus of health monitoring has been shifted even more to the need for self-applicability of sensor systems. In addition to enabling repeated measurements and long-term monitoring, systems suited for self-application add more possibilities to reach remote, bedridden, or quarantined individuals. Self-application also adds convenience for the patient and can therefore increase compliance with the procedure, especially if multiple recordings are planned (Mikkelsen et al., 2019). Ecological validity is further increased because patients can implement the preparation of the measurement into their nighttime routine and the quality of sleep is known to be better when individuals can sleep in familiar environments (e.g., Tamaki et al., 2016).

In this paper, we will focus on two aspects of self-application, data validity and signal quality. Data validity concerns the prospect of deriving information suitable for the relevant interpretation or diagnosis from the data. Signal quality concerns all aspects regarding the usefulness of the data in general, including impedance levels, the number of channels that delivered stable recordings and the signal-to-noise ratio. Sleep staging, for instance, can only be achieved if electrodes are placed at appropriate scalp positions (data validity) and the recorded data offers sufficient information for an adequate interpretation (signal quality). In a first feasibility study, we validate adequate electrodes positioning on the scalp for sleep staging. In a second study, participants were instructed to find the correct positioning of a sensor array themselves. This latter aspect of self-application concerns signal quality in particular. Previous EEG sensors designed for self-application have used dry electrodes (i.e., Arnal et al., 2019). While dry electrodes require no or little skin preparation and can be re-arranged, which makes them convenient for self-application, they require physical pressure to hold electrodes in place, causing discomfort, headaches and sleep disruption. In laboratory settings, wet electrodes remain the gold standard when the aim is to collect data with optimal signal quality. Professionals use wet electrodes in combination with different preparation techniques (skin cleansing and abrasion) and substances (conductive gels or pastes, special adhesives). In the context of self-application, individuals must be trained to perform these or similar steps themselves.

**FIGURE 1:**
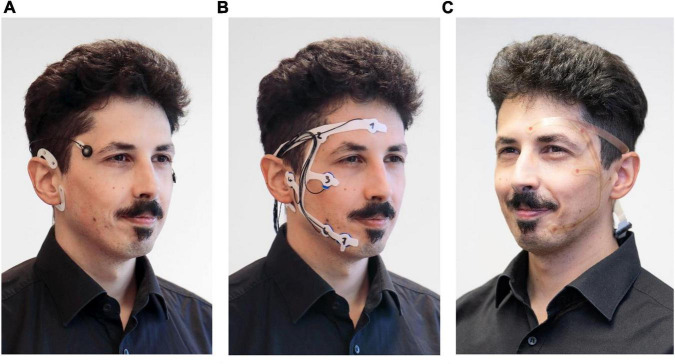
Development steps of the self-applicable, pre-gelled *trEEGrid*. **(A)** stage 1: cEEGrid + EOG, da Silva Souto et al. (2021). **(B)** stage 2: foam *trEEGrid*, current study. **(C)** stage 3: *trEEGrid* prototype on a flexible PCB, future development. ©Fraunhofer IDMT/Anika Bödecker.

Previously, we showed that a system consisting of a single flexible electrode patch around the ear, a cEEGrid (Debener et al., 2015), delivers enough information for a satisfactory sleep analysis (da Silva Souto et al., 2021). However, results were significantly more reliable if EOG information from two electrodes near the eyes were included in the information available to the sleep analyst. Based on these results, we designed a new electrodes array to include positions behind the ear, around the eye and on the chin, for additional EMG acquisition (Figure 1). The new configuration omitted the area below the ear in favor of a bridge above the ear to comfortably connect electrodes from the facial area to those located behind the ear. Preliminary exploration revealed that this design reduced discomfort that can be associated with talking or eating with cEEGrids. Furthermore, this bridge facilitates self-application, as the ear provides additional temporary support. A near-ear electrode in the facial area close to the tragus substitutes the bottom end electrode of the cEEGrid. Concerning grapho-elements, our previous study showed that sleep spindles and K-complexes recorded at Fpz are best represented in cEEGrid channel combinations that point in the direction of Fpz. Here, we expanded the principle of linear combinations by configuring facial electrodes to allow for directional analysis pointing not only to frontal areas, but to occipital and central areas as well. This allowed us to focus on frontal areas that are especially relevant for sleep staging but also offer applications beyond sleep.

In the first study, we validated a miniaturized EEG setup consisting of the new electrode constellation and a wearable amplifier against a commercial mobile PSG system (*validation study*). In the second study, we tested the self-applicability of this system by giving it to participants and instructing them remotely to prepare the sensors for a measurement (*self-application study)*.

## Materials and Methods

### Measurement Systems

The new electrode grid introduced will be referred to as *trEEGrid* system and was used in combination with a wireless EEG amplifier (Smarting Sleep, mBrainTrain, Serbia). The *trEEGrid* is based on experience with the cEEGrid ear-EEG array (Figure 1A), but specifically designed to foster self-application for sleep EEG acquisition at home. In study 1, the general design is tested using a prototype built from pre-gelled neonatal ECG electrodes. The electrodes were embedded within a self-adhesive grid shape using a medical foam plaster (Figure 1B). This prototype helped to validate finalize the *trEEGrid* design, as shown on a PCB grid in Figure 1C. We refer to this intermediate design as foam *trEEGrid.* It included nine single-use self-adhesive gel electrodes (seven channels for EEG, EOG and EMG analysis, reference and ground).

The new prototype was validated by concurrent recordings with commercial PSG system (SOMNOscreen Plus, SomnoMedics, Germany; further referred to as *PSG system*). The commercial system included six EEG electrodes, two piezosensoric belts for thorax and abdomen expansion and a finger clip sensor, which measured oxygen saturation and pulse. The two EMG electrodes of the *PSG system* were not used, since they competed with the space of the *trEEGrid* EMG channels R6 and R7. Electrodes were gold-plated cup electrodes mounted with adhesive gel.

### Participants

Twenty healthy individuals participated in the *validation study*. Eight datasets had to be excluded. Six datasets were excluded due to technical problems concerning the *trEEGrid*, one further dataset due to instability of the Bluetooth connection to the amplifier, and another dataset due to power failure of the SOMNOscreen *PSG system*. The 12 participants (three females, nine males, mean age = 28.9 years, range = 18–45 years) entering the analysis reported no sleep disorders. Each participant provided one night of sleep data, recorded during sleep at home.

Twelve further individuals participated in the *self-application study* (four females, eight males, mean age = 28.8 years, range = 18–55 years). No participant had prior experience in either handling or applying the foam *trEEGrid*, nor was involved in its design process. All participants followed instruction for self-applicating the *trEEGrid* system described above and succeeded in providing a 30 min EEG recording.

For both studies, written consent was given by all participants. Both studies were approved by the local ethics board.

### Data Acquisition

#### Validation Study

For the *validation study*, participants came into the lab in the early evening to be prepared for the study. After given written consent, they were outfitted with the two systems, the commercial *PSG system* and the *trEEGrid* system.

The sensors of the *PSG system* (six EEG electrodes, two piezosensoric belts for thorax and abdomen expansion and a finger clip sensor for oxygen saturation and pulse measurement) were connected to a recording box worn in front of the torso on a belt. EEG recording was sampled at 256 Hz with a reference at Cz and ground at Fz.

**FIGURE 2:**
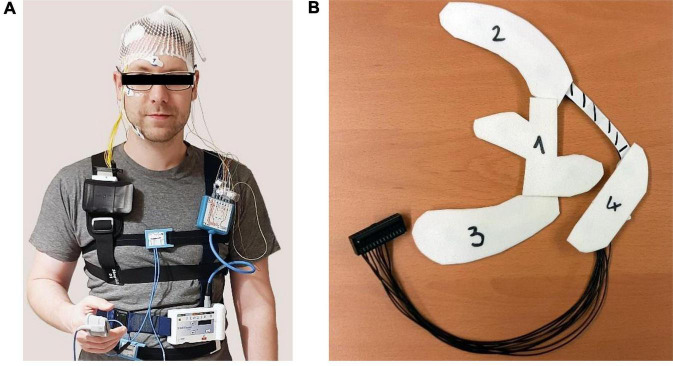
**(A)** Recording setup of the *validation study* including the commercial at-home *PSG system* and the *trEEGrid* system worn at the same time. **(B)** View on foam electrode grid prepared for self-application with wax paper covers in place to guide the participant through application.

The *trEEGrid* electrodes were connected via cable to a smarting amplifier worn in a pouch on a shoulder strap and recording at a sampling rate of 250 Hz to a raspberry pi-based recording station via Bluetooth (see Figure 2A for setup). Participants were then sent home to sleep. The commercial system started recording at a pre-set time indicated as just prior to bedtime by the participant. The *trEEGrid* system was manually started by the participant on a smartphone app before turning off the light. Impedance current was active during recording for continuous impedance measurements. The next morning, participants manually stopped the recording and took off both systems without assistance.

#### Self-Application Study

For the *self-application study*, participants either picked up the materials for the study and joined the online, supervised instruction session from home (10 participants) or they were placed alone in a room at the institute with materials and joined the online, supervised instruction session on a laptop set up for them (two participants). Materials included the *trEEGrid* system, consisting of the self-applicable electrode grid and the amplifier already attached to the chest belt, a mirror, and four cotton pads soaked in alcohol. The *trEEGrid* system was prepared for use by removing adhesives from both the electrodes and the foam grid structure and replacing it by four fitted pieces of wax paper numbered 1 through 4 in the order of the planned removal (see Figure 2B). The experimenter started the online session at the appointed time and taught the participants through a detailed picture presentation consisting of 22 slides accompanied by oral instruction how to self-apply the electrode grid, leaving room for individual questions if needed. In order, participants were instructed to put on the chest belt, prepare the skin, remove the pieces of wax paper and self-apply the electrode grid section by section and connect the grid to the amplifier. Time was recorded for this procedure. Participants filled in a questionnaire, rating statements concerning aspects of self-applicability on an agreement scale of 1 to 5 (1 = “very much,” 2 = “rather,” 3 = “neutral,” 4 = “slightly,” 5 = “not at all”) and answered additional questions in a free text format upon completion of the study.

### Data Analysis of Validation Study

#### Data Preprocessing

Data from both systems (*trEEGrid* and *PSG system*) were prepared for the annotation and the correlation analysis as followed.

First EEG signals were bandstop filtered from 45 to 55 Hz, (phase true, 4th order Butterworth) to reduce the 50 Hz line noise. EEG data of *trEEGrid* was additionally bandstop filtered from 60 to 65 Hz, (phase true, 4th order Butterworth) to reduce impedance current. EEG data of *trEEGrid* and *PSG system* were bandpass filtered from 0.5 to 40 Hz, (phase true, 4th order Butterworth) to reduce electrode drift and noise containing high-frequency components. Due to the Bluetooth connection used in the *trEEGrid* system, its data was checked for package loss using the recorded package number information. If necessary, zeros were added where packages were lost (overall less than 0.0007% of all packages were lost). Data of both systems were downsampled to 125 Hz and then synchronized by aligning ten eyeblinks at the start and end of each measurement. The excess samples before the first and after the last aligned eyeblink were discarded. If there was a size difference of both datasets due to diverging system clocks, *PSG system* data was resized to fit to *trEEGrid* data.

**FIGURE 3:**
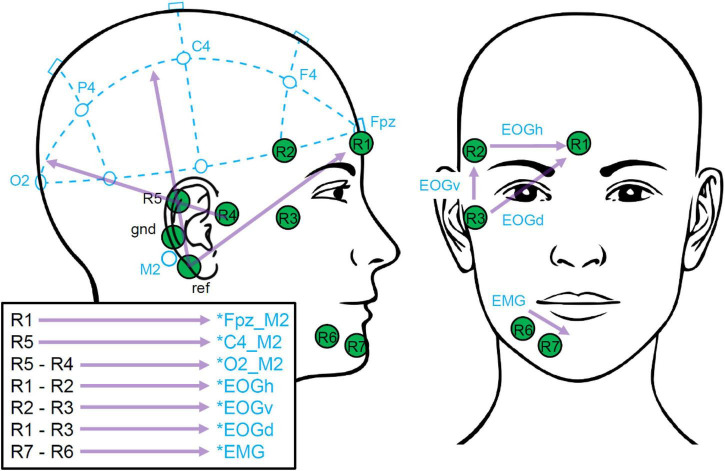
*trEEGrid* channel combinations. These bipolar channels extrapolate toward classical PSG scalp positions (Fpz, C4, O2) as well as EOG and EMG, as illustrated.

Channels of both datasets were selected, re-referenced and labeled as listed in Table 1. For the *trEEGrid* layout, linear combinations of channels were used. This step was motivated to approximately represent the EEG measured at classic PSG-relevant scalp positions Fpz, C4 and O2 when referenced to the right mastoid process M2 (in the following marked with an asterisk, as shown in Figure 3).

**TABLE 1:** Channel configuration of the *trEEGrid* and *PSG system* data sets.

Data set	*trEEGrid*	PSG system
EEG channels	R1 ➔ *Fpz_M2	F3_M2
	R5 ➔ *C4_M2	F4_M1
	R5-R4 ➔ *O2_M2	C3_M2
		C4_M1
		O3_M2
		O4_M1

EOG	R1-R2 ➔ *EOGh	E1_M2
	R2-R3 ➔ *EOGv	E2_M1
	R1-R3 ➔ *EOGd	

EMG	R7-R6 ➔ *EMG	None

Additional sensors	None	SPO2
		Puls
		Pleth
		RIP_Thrx
		RIP_Abdom
		Summe_RIPs

The symbol * is used to differ the names of the channel-combinations from ones of the classical scalp positions.

*This is done because the channel-combinations are used as an estimation of the EEG that could be measured at those classical scalp positions.*

#### Data Annotation of the Expert Scorer

An expert polysomnographic technologist with 15 years of experience polysomnography (MP, subsequently referred to as expert scorer) annotated the sleep data of both systems independently, by evaluating consecutive 30 s segments. Participant labels of both systems were randomized independently prior annotation to lower a possible bias by the expert scorer. An open source, sleep analysis software was used for this purpose (Combrisson et al., 2017). Sleep staging was done in accordance with AASM guidelines [annotated stages: awake (W), N1, N2, N3 and REM (rapid eye movement)]. In addition, sleep spindles were annotated in EEG data of the *PSG system*.

#### Statistical Analysis

To statistically evaluate differences of the impedance measurements, a repeated analysis of variance (rANOVA) was done. To test for sphericity of the data a Mauchly-Test was applied. In case of significance, results were Greenhouse-Geisser corrected.

To statistically evaluate the accordance of the different hypnograms, Cohen’s Kappa was used to determine inter-rater reliability (agreement scale according to Landis and Koch, 1977). For a calculation of the reliability over participants, the corresponding hypnograms were concatenated. To this end, confusion matrices of the different hypnograms were calculated and used to determine Cohen’s Kappa. Cohen’s Kappa was calculated for every single sleep stage vs. the rest, and across all sleep stages.

#### Correlation Analysis

da Silva Souto et al. (2021) showed that sub-hairline EEG electrodes can be used to estimate the EEG measured at further distanced location of the scalp. The authors correlated sleep spindles annotated at a classical Fpz electrode with corresponding EEG recordings of linear combinations of ear electrodes and showed that highest correlations were scored at channel combinations that point in the direction of Fpz. To further explore this approach, we expanded the analysis to central and occipital PSG locations of the scalp. Therefore, we first extracted spindle events by separating of the transient EEG of the *trEEGrid* channels (R1, …, R7) as well as the transient EEG of the PSG channels F4_M2, C4_M2 and O2_M2 into epochs according to the sleep spindle annotations of the expert scorer in *PSG system* rating. The annotated epochs of each channel F4_M2, C4_M2 and O2_M2 are used as reference signals in three appropriate test conditions of the correlation analysis. DC-offset was reduced by subtracting the average over all samples of the respective epoch. Finally, correlation coefficients were calculated between the epochs of the three reference signals and the respective transient data of all possible single channel and bipolar channel combinations of *trEEGrid* electrodes (28 in total) as shown in the following:


$$\begin{array}{ccccccc} R\,1, & & & & & & \\ R\,1\,\text{-}\,R\,2, & R\,2, & & & & & \\ R\,1\,\text{-}\,R\,3, & R\,2\,\text{-}\,R\,3, & R\,3, & & & & \\ R\,1\,\text{-}\,R\,4, & R\,2\,\text{-}\,R\,4, & R\,3\,\text{-}\,R\,4, & R\,4, & & & \\ R\,1\,\text{-}\,R\,5, & R\,2\,\text{-}\,R\,5, & R\,3\,\text{-}\,R\,5, & R\,4\,\text{-}\,R\,5, & R\,5, & & \\ R\,1\,\text{-}\,R\,6, & R\,2\,\text{-}\,R\,6, & R\,3\,\text{-}\,R\,6, & R\,4\,\text{-}\,R\,6, & R\,5\,\text{-}\,R\,6, & R\,6, & \\ R\,1\,\text{-}\,R\,7, & R\,2\,\text{-}\,R\,7, & R\,3\,\text{-}\,R\,7, & R\,4\,\text{-}\,R\,7, & R\,5\,\text{-}\,R\,7, & R\,6\,\text{-}\,R\,7, & R\,7. \end{array}$$

If a *trEEGrid* channel impedance exceeded 60 kΩ during an annotation window, the results of this channel and all related channel combinations were discarded for this specific epoch (13.6% epochs rejected on average for all listed combination and participants). To calculate the median correlation coefficient over epochs or over participants, the single correlation coefficients were first Fisher Z transformed than the median was formed. In a final step, its result was transformed back, by calculating its hyperbolic tangent. The transformation is necessary to approximate the normal distribution of correlation coefficients and reduce the bias of their average (Silver and Dunlap, 1987).

## Results

### Validation Study

#### Channel Impedances

Table 2 shows impedance values at 5 min after the start and before the end of the night measurements. It is noticeable, that chin electrodes (R6 and R7) lost connection during the measurement for six out of 12 participants and the electrode in front of the ear (R4) for four out of 12 participants. In 11 out of 15 cases the loose electrodes reconnected overnight. Overall impedances dropped significantly at the end of the night [*F*(1,11) = 11.8; *p* < 0.01]. Across electrodes there was a significant difference of impedance [*F*(6,66) = 5.1; *p* < 0.01], but no significant difference across electrodes and overnight combined [*F*(6,66) = 2.3; *p* = 0.08].

**TABLE 2:** *trEEGrid* system impedances at the start and end of night measurements in the *validation study* in kΩ (impedances above 100 kΩ are marked as “-”).

Participants	Impedance measurements [kΩ]													
	R1		R2		R3		R4		R5		R6		R7	
	Start	End	Start	End	Start	End	Start	End	Start	End	Start	End	Start	End
**P1**	7.2	11.7	17.9	15.5	10.9	10.0	–	45.1	16.1	10.8	–	3.9	8.1	8.3
**P2**	13.1	14.0	14.5	11.8	40.1	25.3	–	22.8	55.2	44.1	–	36.7	–	34.5
**P3**	12.4	9.7	38.4	26.3	19.6	13.3	36.6	28.5	93.2	54.0	23.0	16.5	21.6	12.3
**P4**	17.4	21.9	18.5	24.4	22.8	5.1	24.6	34.2	18.4	17.7	18.1	25.3	17.6	19.1
**P5**	18.8	13.2	29.4	11.8	28.6	24.4	51.8	19.5	39.2	19.9	14.7	12.8	22.3	–
**P6**	55.1	25.0	52.2	22.5	56.3	51.5	76.8	54.0	62.5	31.9	–	–	–	–
**P7**	13.1	10.9	16.5	13.0	25.8	17.6	–	18.2	30.4	21.3	–	–	–	–
**P8**	11.6	12.2	16.2	12.5	15.1	14.4	18.9	12.0	4.9	5.0	27.2	41.9	20.4	12.5
**P9**	24.1	37.9	33.2	33.2	21.7	13.1	–	36.3	53.5	40.3	–	48.2	52.1	28.5
**P10**	11.0	15.1	15.0	–	15.0	14.3	27.7	23.9	–	31.5	15.8	16.4	26.4	24.6
**P11**	7.6	6.9	13.0	9.9	21.7	18.9	40.3	42.8	10.8	11.1	13.6	10.5	11.0	10.0
**P12**	14.0	9.9	18.6	9.2	36.7	26.2	12.2	11.9	66.6	26.9	–	34.9	–	54.6

#### Analysis of Hypnograms

**FIGURE 4:**
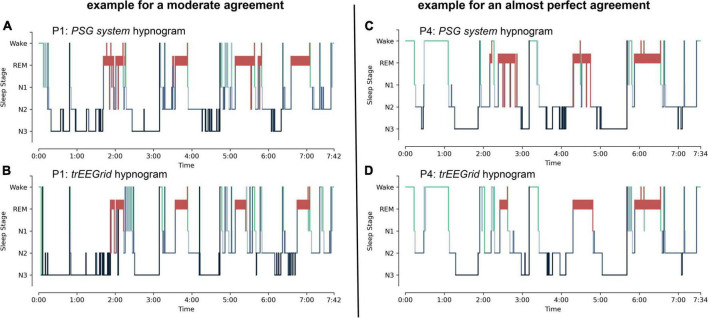
Exemplary hypnograms, shown for participants P1 and P4. **(A,B)** Hypnograms of P1 show a moderate agreement. **(C,D)** Hypnograms of P4 show an almost perfect agreement. The sleep stages are defined according to the AASM as: Art (Artifact), Wake (Wakefulness), REM (Rapid Eye Movement), N1 (non-REM1), N2 (non-REM2) and N3 (non-REM3).

Hypnograms of both systems are shown in Figure 4 for two participants exemplarily (P1 and P4). The lowest reliability tests result of a single participant was scored for P1 (shown in Figures 4A,B) with Cohen’s κ = 0.58 ± 0.02 (moderate agreement). The highest tests result was scored for P4 (shown in Figures 4C,D) with Cohen’s κ = 0.83 ± 0.01 (almost perfect agreement). All single participant test results are shown in Table 3.

**TABLE 3:** Statistical reliability for individual participants (standard error ± 0.02).

Participant	P1	P2	P3	P4	P5	P6	P7	P8	P9	P10	P11	P12
**Cohen’s κ**	0.58	0.75	0.77	0.83	0.65	0.60	0.77	0.83	0.70	0.58	0.76	0.64

The confusion matrix corresponding to hypnograms of all participants and systems combined is shown in Table 4. The respective statistical reliability test results over all participants and corresponding standard errors are shown in the following for either every single sleep stage vs. the rest or all sleep stages combined:

**TABLE 4:** The hypnograms of all participants respective to each system were concatenated to create this confusion matrix.

*trEEGrid* vs. PSG system	Art	W	N1	N2	N3	REM
**Art**	0	0	0	0	0	5
**W**	2	934	110	40	10	15
**N1**	1	175	476	159	14	32
**N2**	8	90	204	2,832	345	92
**N3**	0	15	14	495	2,620	4
**REM**	0	88	222	219	24	1,387

*Each count refers to the annotation of a single 30 s segment.*

Wake: Cohen’s κ = 0.75 ± 0.01 (substantial agreement).

N1: Cohen’s κ = 0.46 ± 0.02 (moderate agreement).

N2: Cohen’s κ = 0.66 ± 0.01 (substantial agreement).

N3: Cohen’s κ = 0.79 ± 0.01 (substantial agreement).

REM: Cohen’s κ = 0.76 ± 0.01 (substantial agreement).

All: Cohen’s κ = 0.70 ± 0.01 (substantial agreement).

#### Correlation Analysis

**FIGURE 5:**
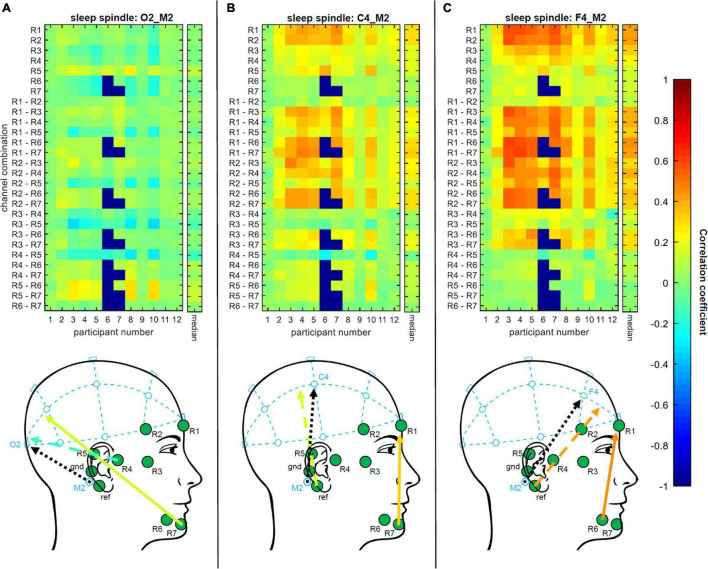
**Top:** Single participant and median results of all three conditions of the correlation analysis. Correlation coefficients between sleep spindles annotated at EEG of PSG system (using **(A)** O2_M2, **(B)** C4_M2 and **(C)** F4_M2) and the corresponding EEG of every possible single channel and bipolar channel linear combinations of *trEEGrid* electrodes are calculated, Fisher Z transformed, formed the median over epochs and participants, and transformed back. **Bottom:** Visual comparison of alignment of *PSG system* channels (dotted black arrows: O2_M2, C4_M2 and F4_M2) and corresponding *trEEGrid* channel combinations that scored highest median correlation coefficients (solid colored arrows). Feasible linear combinations of *trEEGrid* channels placed around ear are shown for each condition (dashed colored arrows). Colors represent the magnitude of correlations. Based on the correlation analysis *trEEGrid* channel linear combination that either point into the direction of O2_M2, C4_M2 and F4_M2, respectively, or are in parallel to its alignment, result in highest absolute correlation (negative correlations point in the opposing direction of the channel combination) and therefore are best suited to represent sleep spindle reference measurements of the *PSG system* (O2_M2, C4_M2 and F4_M2).

**FIGURE 6:**
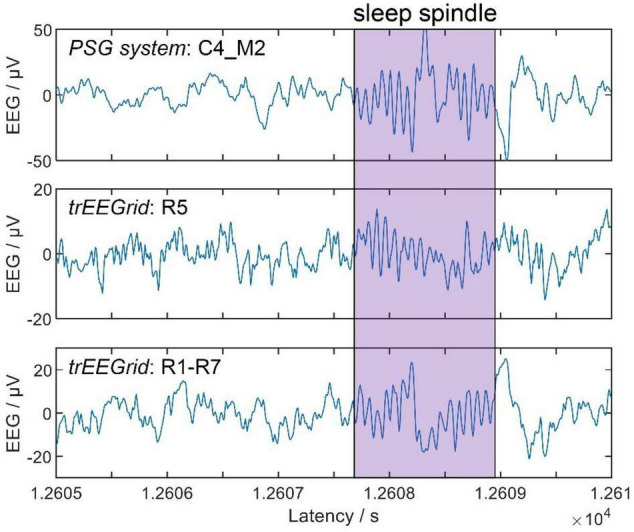
Example showing a single annotated sleep spindle of participant P4, captured by the *PSG system* (**top**, channel C4_M2) and *trEEGrid* channels R5 **(middle)** and R1-R7 **(bottom)**.

Figure 5 (top) shows the results of all three test conditions of the correlation analysis. It should be noted that all annotations of channel R6 and all related channel combinations were discarded for participant 7 as well as channel R7 and all related channel combinations for participant 7, since channel impedance exceeded 60 kΩ at all annotation windows. In all three conditions, clusters of *trEEGrid* channel combinations are visible that have similar absolute correlation coefficients. Overall, R5-R7 and R4-R5 scored the highest absolute correlations to sleep spindles measured at O2_M2 of the *PSG system* (0.15 and −0.14), R1-R7 and R1-R6 the highest to C4_M2 (0.32 and 0.31) and finally R1-R6 and R1 the highest to F4_M2 (0.46 and 0.43). In all three conditions, it is noticeable that sleep spindles measured at O2_M2, C4_M2 and F4_M2 are best represented in EEG of *trEEGrid* channel linear combinations that either point into the direction of the respective *PSG system* electrodes, or are in parallel to its alignment [as shown in Figure 5 (bottom)]. This result is exemplary shown in Figure 6, by comparing a single annotated sleep spindle of participant P4 recorded at C4_M2 of the *PSG system* with the EEG recorded at R5 and R1-R7 of the *trEEGrid*.

These results further confirm the assumption made in da Silva Souto et al. (2021) that combinations of ear EEG channels can be used to estimate the EEG measured at distant locations of the scalp.

### Self-Application Study

**FIGURE 7:**
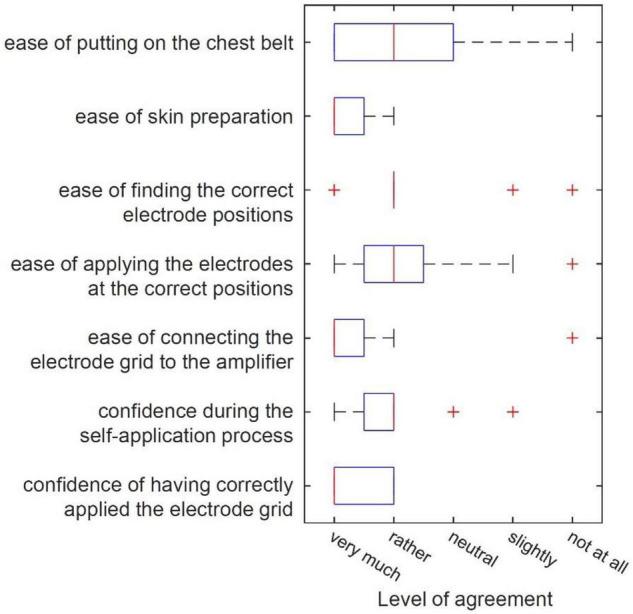
Box plots of answers given by the 12 participants of the *self-application study* concerning different aspects of self-applicability.

Figure 7 shows the answers to the self-applicability questionnaire in box plots. Seven out of 12 participants answered the question “How easy was it to put on the chest belt?” with “very much” or “rather.” All 12 participants answered the question “How easy was it to prepare the skin?” with “very much” or “rather.” 10 out of 12 participants answered the question “How easy was it to find the correct electrode positions?” with “very much” or “rather.” Nine of 12 participants answered the question “How easy was it to apply the electrodes at the correct positions?” with “very much” or “rather.” 10 out of 12 participants answered the question “How easy was it to connect the electrode grid to the amplifier?” with “very much” or “rather.” 10 out of 12 participants answered the question “How confident did you feel during the self-application process?” with “very much” or “rather.” All 12 participants answered the question “How confident are you of having applied the electrode grid correctly?” with “very much” or “rather.” Asked for the difficulties experienced during the self-application process in a free text format, five participants mentioned the positioning behind the ear as giving them the most difficulty, one mentioned difficulty with using the mirror image, one mentioned the order of application of the different electrodes, and one noted that self-applying alone without assistance was the most difficult part.

Table 5 shows the set-up time of each participant in minutes. On group average, the mean set up time was 12 min (rounded to a 30 s interval) with a range of 8:00 to 19:30 min. The impedance values after 20 min of wear ranged between 5.1 and 98.6 kΩ, excluding values above 100 kΩ. Comparing overall impedances at the start of the overnight measurement of the *validation study* with the recorded impedances of the *self-application study* showed no significant difference [*F*(1,11) = 1.3; *p* = 0.28]. Across electrodes there was a significant difference of impedance [*F*(6,66) = 9.1; *p* < 0.01], and a significant difference across electrodes and between the two measurements combined [*F*(6,66) = 3.7; *p* = 0.04]. Importantly, this interaction is not driven by impedance differences between the two measurements at any electrode, but rather by divergent differences between electrodes within each measurement.

**TABLE 5:** Results of *self-application study* measurements.

Participants	Setup time[min]	Impedance after 20 min [kΩ]						
		R1	R2	R3	R4	R5	R6	R7
**SA01**	14:30	26.3	–	23.9	86.5	70.3	15.1	42.9
**SA02**	11:30	59.2	67.5	30.3	98.6	92.2	35.4	59.3
**SA03**	08:00	6.5	25.5	23.7	29.7	34.5	35.9	–
**SA04**	13:00	18.3	44.3	17.4	9.7	52.4	16.7	5.1
**SA05**	11:00	–	–	–	–	–	–	–
**SA06**	11:00	8.9	7.1	10.5	43.1	42.6	31.7	–
**SA07**	14:00	23.2	45.7	36.0	69.1	–	11.9	9.5
**SA08**	19:30	19.9	16.9	27.6	35.6	–	10.0	5.5
**SA09**	10:00	19.7	61.4	23.6	–	–	–	–
**SA10**	09:30	8.5	27.6	13.3	27.8	–	15.6	–
**SA11**	15:00	18.1	28.2	9.9	–	–	22.1	13.3
**SA12**	09:30	24.3	73.6	21.4	25.5	91.4	16.0	19.3

*For each participant the time needed for self-application of the trEEGrid is given in minutes (rounded to 30 s intervals).*

*Impedance values measured after 20 min of wear are given in kΩ (impedances above 100 kΩ are marked as “-”).*

## Discussion

In the present study we tested the feasibility of a new sensory grid design (referred to as *trEEGrid*) consisting of nine pre-gelled ECG electrodes around the eye, ear and chin for standard sleep staging recordings. The *trEEGrid* was used in a mobile setting and validated by comparing it to concurrent recordings obtained from a commercial *PSG system*.

Overnight impedance measurements of the *trEEGrid* showed that pre-gelled ECG electrodes were feasible for sleep EEG measurements. Further it was interesting that in 11 out of 15 cases ECG electrodes that lost connection at the start of the measurement recovered connection during the night. This could be a benefit of pre-gelled ECG electrodes over conventional electrodes using electrolyte gels that solidify over time. In this case, recovery of the connection would not be possible after breakage of the solidified gel.

We compared hypnogram annotations of both systems by calculating inter-rater reliability values and found, overall, a substantial agreement between both systems for all participants. Further reliability tests of single sleep stages showed agreements comparable to the literature (Danker-Hopfe et al., 2009), and very similar to previous ear-EEG sleep studies (i.e., Sterr et al., 2018). It must be taken into account that the signals based on the linear combinations of the *trEEGrid* are similar but still different to the signals of the classical EEG electrode positions. This poses a special challenge to the expert scorer, who is used to the classical setting. Automated sleep staging approaches based on machine learning could possibly achieve even better results, in particular when approaches are trained on *trEEGrid* data.

We compared sleep spindles annotated in the *PSG system* with the respective recordings of different *trEEGrid* channel linear combinations in a correlation analysis. In agreement with da Silva Souto et al. (2021), the results provide further evidence that linear combinations of ear EEG channels can be used to extrapolate information measured at traditional locations on the scalp, like O2 and C4. This motivates the choice of linear combinations of *trEEGrid* channels used for hypnogram annotations. A preliminary test showed that it is possible for the expert scorer to annotate sleep spindles in the EEG recording of the *trEEGrid*. In a future study it would be interesting to analyze if these annotations would differ from those made in a conventional *PSG system*.

We showed that the *trEEGrid* system is self-applicable by healthy participants with only remote instruction. Participants had little trouble finding the correct positions and applied the self-adhesive grid to facial areas adequately. Note that self-application was supervised, so the experimenter was able to give feedback during the process. In a real-life scenario, the goal would be to offer a system that does not rely on an additional person guiding the process. Also, participants in this study were predominantly young adults who had no motor, visual, or hearing impairments that hindered their ability to understand and follow the instructions. For applications that focus on diagnosing or monitoring disorders associated with sleep, older adults may be more afflicted and therefore make up a larger percentage of the user base. In half of the participants, some electrodes lost connection during the measurement and prevented adequate data acquisition. This happened more often with chin electrodes than with any other electrodes. Reasons included movement from eating and talking after applying the grid or insufficient adhesion on a bearded face. Timing the application of the sensors closer to bedtime for a sleep measurement may help to keep sensors in place for a longer period of time, though one participant reported loosening of a chin electrode within 30 min of application.

The overall comparison of impedance values between experimenter-application and self-application showed no significant difference. One major challenge in the application of the *trEEGrid* seemed to be electrode R5, sitting at the top behind the ear. In the *validation study*, this channel lost connection only for one out of 12 participants; in the *self-application study*, however, this channel remained out of range for six out of 12 participants. The position R5 poses a challenge by being both visually occluded and not completely hairless for most participants, therefore rendering it difficult to achieve good signal quality without assistance.

Remarkably, most participants found the electrode grid to be sized exactly right for their faces. Although we did not collect data on facial metrics of the participants, human heads come in different shapes and sizes. The prototype used in the studies was built of single pre-gelled electrodes fixed into a foam grid for exact placement and additional adhesion. The flexibility of the foam material allowed participants to place sensors where they needed to be while still using the grid shape as a guide. This feature of the grid prototype will be translated into future versions which should be stretchable PCB based as shown in Figure 1C, allowing the stretchiness and flexibility of a foam grid while being lighter and smaller.

In the past few years, a variety of new materials for biosignal detection have been introduced which offer fascinating possibilities for face application, including facial tattoos (Shustak et al., 2018), dry film electrodes that rely on surface tension caused by the person’s sweat for adhesion (Nawrocki et al., 2018) or conductive aerogel film (Saadatnia et al., 2021). These sensor materials have in common that multiple sensor location can be arranged on a grid which replaces the need for cables and acts as a guide for correct placement at the same time.

In conclusion, the use of self-applicable, comfortable and discrete sensor solutions around the ear could advance future sleep diagnostics by facilitating repeated home-sleep EEG acquisition. Beyond sleep, future disposable flex-printed arrays could be made out of stretchable material, thereby further increasing wearing comfort and signal quality. We argue that improving wearing comfort and unobtrusiveness of sensors is of crucial importance for achieving hassle-free, long-term EEG monitoring solutions, which are of value in health screenings and at-risk neurological and psychiatric patients (Fernandez and Lüthi, 2020; Mander, 2020).

## Data Availability Statement

The raw data supporting the conclusions of this article will be made available by the authors, without undue reservation.

## Ethics Statement

The studies involving human participants were reviewed and approved by Research Ethics Committee, University of Oldenburg, Oldenburg, Germany. The participants provided their written informed consent to participate in the study. Written informed consent was obtained from the individual(s) for the publication of any potentially identifiable images or data included in this article.

## Author Contributions

CS, WP, and KW designed the experiments. WP and CS collected the data. MP scored all the sleep data and coded grapho-element annotations. CS and WP analyzed the data. SD provided feedback during all the stages of conducting the studies. WP, CS, SD, and KW wrote the manuscript. All authors contributed to the article and approved the submitted version.

## Conflict of Interest

The authors declare that the research was conducted in the absence of any commercial or financial relationships that could be construed as a potential conflict of interest.

## Publisher’s Note

All claims expressed in this article are solely those of the authors and do not necessarily represent those of their affiliated organizations, or those of the publisher, the editors and the reviewers. Any product that may be evaluated in this article, or claim that may be made by its manufacturer, is not guaranteed or endorsed by the publisher.
